# Effect of Supplementing a Cricket Diet with Cooked Green Beans, a Discarded Agro-Industrial Material, on Performance of *Gryllus madagascarensis* at Two Rearing Densities

**DOI:** 10.3390/insects17040411

**Published:** 2026-04-10

**Authors:** Tahiry M. Raharimandimby, Tanjona Ramiadantsoa, Hans C. Kelstrup, Sylvain Hugel, Brian L. Fisher

**Affiliations:** 1 Madagascar Biodiversity Center, Tsimbazaza, Antananarivo 101, Madagascarbfisher@calacademy.org (B.L.F.); 2 Institut des Neurosciences Cellulaires et Intégratives, Centre National de la Recherche Scientifique, Université de Strasbourg, 67000 Strasbourg, France; 3 Department of Entomology, California Academy of Sciences, San Francisco, CA 94118, USA

**Keywords:** edible crickets, feed supplementation, insect diet, sustainability, circular food system

## Abstract

This study investigated whether cooked green beans, a discarded agro-industrial material, can partially replace commercial chicken feed to improve the performance of the edible cricket *Gryllus madagascarensis*, while accounting for the strong effects of rearing density. A two-by-two factorial experiment was conducted in which crickets were reared at low (500 individuals per box) or high density (2500 individuals per box) and fed either standard chicken feed or the same feed supplemented with cooked green beans, with twenty replicates per treatment. Survival, chicken feed consumption, biomass yield, frass production, efficiency of conversion of ingested feed, and approximate digestibility were measured over the rearing period. Survival increased from 30.0% to 32.9% at low density and from 11.7% to 13.2% at high density, while biomass yield increased from 117.2 g to 129.7 g and from 194.4 g to 231.2 g, respectively. Frass production also increased under supplementation. In contrast, individual body weight was not improved, indicating that higher biomass production resulted mainly from increased survival rather than faster growth. Rearing density remained a major determinant of performance, with low density favoring individual size and survival, and high density maximizing total biomass. Overall, partial replacement of commercial feed with cooked green beans improved survival, biomass yield, and frass production, supporting the use of plant-based wastes to enhance the sustainability of cricket farming.

## Simple Summary

Edible crickets are increasingly farmed as a sustainable source of protein, but feed costs and waste management remain major challenges for producers. This study tested whether cooked green beans, a discarded agro-industrial material, could partly replace commercial chicken feed to improve cricket production. Crickets were raised at low and high stocking densities and fed either standard feed or supplemented with cooked green beans. Adding green beans increased survival and total cricket biomass at both densities, especially when crickets were kept at high density. However, individual crickets did not grow larger, showing that higher production mainly resulted from more crickets surviving rather than faster growth. Green bean supplementation also increased the production of cricket waste, which can be reused as organic fertilizer. These results show that plant-based wastes such as green beans can improve cricket farming efficiency, reduce reliance on commercial feed, and limit food waste. This approach can help farmers increase production while supporting a more sustainable and circular food system.

## 1. Introduction

Ensuring global food security has become one of the most pressing challenges of the current century. As the human population continues to grow, demand for safe, nutritious, and sustainably produced food is increasing rapidly [1,2]. At the same time, conventional food production systems, largely dependent on livestock farming and large-scale crop cultivation, are increasingly constrained by high resource requirements, land degradation, and substantial greenhouse gas emissions [3,4]. Identifying alternative protein sources that reduce environmental impacts while remaining economically viable at the farm scale is therefore an urgent global priority.

Among the many alternatives explored, edible insects have received growing attention. More than 2100 insect species are known to be edible worldwide [5], and crickets in particular are among the most studied groups for commercial farming. They are listed by the Food and Agriculture Organization (FAO) as suitable for food and feed mass production [6]. In Madagascar, several cricket species are traditionally consumed, and crickets are increasingly recognized for their potential in both small-scale household rearing and industrial production systems [7,8].

Crickets offer multiple advantages that position them as promising sustainable protein sources. They are nutritionally rich, providing high-quality protein, essential amino acids, and micronutrients, and they convert feed into biomass more efficiently than conventional livestock such as poultry, pigs, and cattle [9,10,11,12]. Their production requires relatively little land, water, and feed and generates substantially fewer greenhouse gas emissions [9,13]. Beyond these efficiencies, cricket farming aligns well with circular-economy approaches, as crickets can be reared on a wide range of organic side-streams and agro-industrial by-products, thereby valorizing materials that would otherwise be wasted [14,15,16]. In addition, cricket frass is increasingly recognized as a valuable organic fertilizer that contributes to nutrient recycling and improved soil fertility [17,18].

Among species used in edible-insect production, *Gryllus madagascarensis* Walker, 1869 is particularly well suited to farming in Madagascar. This species has a short development cycle of approximately 28 days, enabling rapid production turnover [19]. It is suitable for both human food products and animal feed formulations, and its natural occurrence in Madagascar reduces reliance on imported species while ensuring adaptation to local environmental conditions.

Despite these advantages, the expansion of cricket farming is often constrained by diet formulation. Feed represents the dominant operational cost in cricket production, and reliance on commercial chicken feed can limit economic viability in systems supplying low-cost protein markets. Although crickets are capable of utilizing a wide range of organic substrates, not all agro-industrial wastes support optimal growth, survivorship, or productivity. Identifying locally available, low-cost feed ingredients that can partially replace commercial feed without compromising performance is therefore critical for improving the sustainability and scalability of cricket farming systems. This challenge is especially relevant in Madagascar, where diverse plant-based by-products and discarded materials are available but remain insufficiently evaluated for use in insect production.

In addition to diet composition, rearing density is a key factor shaping cricket performance and production outcomes. High stocking densities are commonly used to maximize output per unit area, yet they can intensify competition, stress, and aggressive interactions, leading to reduced individual growth and survivorship. Understanding how dietary supplementation interacts with rearing density is therefore essential for optimizing both biological performance and farm-level productivity.

In this study, we evaluated the effect of supplementing standard chicken feed with cooked green beans, a discarded agro-industrial material, on the performance of *Gryllus madagascarensis* reared under contrasting stocking densities. Specifically, we asked: (i) whether green bean supplementation increases survivorship, chicken feed consumption, biomass yield, and frass output relative to a chicken-feed-only control; (ii) how these effects depend on rearing density, by comparing low-density (500 crickets per box) and high-density (2500 crickets per box) populations; and (iii) how density and diet jointly influence feed efficiency. By addressing these questions, we aim to assess whether cooked green beans can serve as a cost-effective, locally available supplement to improve productivity and enhance the circularity of cricket farming systems based on *Gryllus madagascarensis*.

## 2. Materials and Methods

### 2.1. Study Animal and Rearing Conditions

One-day-old (less than 24 h) crickets, *Gryllus madagascarensis*, were obtained from the hatchery. They were reared for 28 days at the facility under controlled conditions: temperature of 30.6 ± 1.1 °C, relative humidity of 73.9 ± 3.6%, and a 12 h:12 h light:dark photoperiod. Environmental conditions were monitored continuously and averaged across the rearing period.

### 2.2. Diet Treatments

Two feeds were used during the experiment, chicken feed (control) and cooked green beans (supplement). The control feed consisted of a commercial broiler starter diet (LFL, Antananarivo, Madagascar), routinely used in cricket farming. This feed is characterized by low moisture content and is rich in both protein and carbohydrates [19] (Table 1). The chicken feed was directly supplied with no additional processing. Cooked green beans were obtained from a local food factory (Lecofruit, Antananarivo, Madagascar), a discarded material from canned vegetable production. This feed is characterized by high moisture content and low macronutrient content [20] (Table 1). Cooked green beans were therefore considered primarily a moisture-rich supplement rather than a major nutrient source. Green beans were stored in the fridge at 4 °C. Before supplementation, green beans were equilibrated to rearing temperature (30 °C).

### 2.3. Experimental Design

Crickets were reared following a 2 × 2 factorial design with two rearing densities (500 and 2500 crickets per box) and two diet treatments (chicken feed only and chicken feed supplemented with cooked green beans). This design resulted in four treatment groups with 20 replicates each (total of 80 boxes). The densities were defined based on the expected degree of crowding and competition among individuals. The low-density treatment (500 crickets per box) was expected to provide sufficient space, minimizing physical interactions and competition. Conversely, the high-density treatment (2500 crickets per box) was expected to induce crowding conditions, leading to increased competition for food and microclimatic resources and potentially higher stress levels. These contrasting densities were selected to assess the influence of crowding on cricket growth performance and survival.

The boxes were distributed across three shelving units, each composed of five tiers, with each tier supporting six boxes. To minimize environmental bias, treatments were arranged across tiers according to a predetermined order. To further reduce positional effects, the order of tiers was rotated weekly in a descending manner: the top tier became the second, the second tier became the third, and so on, while the bottom tier was moved to the top position.

Each rearing box consisted of a plastic container (545 × 400 × 330 mm) with ventilation on the lid, five egg cartons stacked on one side of the box, and three plates arranged on the other side. Two of the plates were designated for feed and the third plate was designated for a watering device.

To estimate the initial stocking density, groups of 250 newly hatched crickets (neonates) were counted and weighed using a precision balance (UNIWEIGH UG50, Shanghai, China, readability: 50 g × 0.001 g). This procedure was repeated ten times, yielding the following batch weights: 0.177 g, 0.167 g, 0.150 g, 0.161 g, 0.152 g, 0.149 g, 0.152 g, 0.153 g, 0.148 g, and 0.155 g. The mean weight of a single neonate was obtained by dividing each batch weight by 250 and averaging across all ten batches, resulting in an estimated individual weight of 0.626 ± 0.012 mg. This value was then used to calculate the total mass corresponding to the required number of neonates per replicate. Replicates with 500 neonates per box received approximately 0.313 g of neonates, while replicates with 2500 neonates per box received approximately 1.563 g. The estimated number of introduced neonates varied by an average of 1.3% for low-density and 0.27% for high-density conditions. These percentages were obtained by calculating, for each replicate, the deviation between the estimated and target numbers of neonates (500 or 2500), dividing the absolute deviation by the target number to express it as a percentage, and then averaging these percentage deviations across replicates.

### 2.4. Feeding and Water Provision

Chicken feed was provided ad libitum in shallow, nearly flat plates placed on the surface of each rearing box, allowing the feed to remain spread in a thin layer and limiting displacement into the substrate by cricket activity. Remaining chicken feed was checked daily and replenished when necessary. The total amount of chicken feed provided over the rearing period was recorded, and residual feed was collected and weighed at the end of the experiment to estimate total consumption. Cooked green beans were provided daily in the same plates. Uneaten portions were removed and weighed at each feeding interval before being replaced with fresh material in order to prevent mold proliferation, fly infestation, and feed desiccation. The quantities of green beans provided and removed were recorded daily, allowing precise estimation of their consumption.

Both chicken feed and cooked green beans were supplied daily in quantities adjusted to cricket developmental stages to maintain ad libitum access. The amount of green beans increased progressively over the course of the experiment, from approximately 5 g per box per day at early stages to approximately 50 g at later stages; the exact daily amounts provided to each box are reported in the public dataset. At no point during the experiment were either feed sources completely exhausted between feeding intervals.

In both cases, remaining feed was collected directly from the plates, including fine particles. While minor losses due to fragmentation or accidental displacement cannot be entirely excluded, this approach ensured that most uneaten feed remained recoverable.

Water was supplied using cotton balls moistened with water. The cotton balls were soaked and then gently squeezed to remove excess water, ensuring they remained moist but without visible droplets. They were rehydrated daily to prevent drying.

### 2.5. Harvesting and Measurement

Crickets were harvested on the 28th day of the experiment. For each box, live crickets were weighed using precision balance (RADWAG PS1000.R1, Radom, Poland, readability: 1000 g × 0.001 g). The crickets were then packed in plastic bags and frozen at −20 °C for 48 h prior to counting them. Accumulated frass remaining in the boxes was collected and weighed.

The variables measured in this experiment included survival, chicken feed consumption, biomass yield, and frass output. From these, efficiency of conversion of ingested feed (ECI) and approximate digestibility (AD) were calculated.

Survival was calculated as the final number of live crickets/estimated initial number of crickets per box. Chicken feed consumption was calculated by subtracting removed feed at the end of experiment from provided feed and dividing it by the number of surviving crickets. Biomass yield was measured by weighing live crickets harvested from each box, and body weight was measured as cricket biomass yield/surviving crickets. Frass output was measured by weighing cricket droppings collected from each box; care was taken to exclude any uneaten feed residues from the measurement. Efficiency of Conversion of Ingested Feed (ECI) was calculated as the ratio of biomass gain to chicken feed consumed per surviving cricket, i.e., individual weight/feed consumed. Approximate Digestibility (AD) was calculated as the proportion of chicken feed consumed that was not egested as frass per surviving cricket, i.e., (feed consumed − frass output)/feed consumed [21]. Green beans were excluded from both ECI and AD calculations due to their negligible nutritional contribution and high-water content.

### 2.6. Data Analyses

Statistical analyses were performed in R version 4.4.2 (R Core Team, 2024 [22]) using the packages ‘tidyverse’, ‘car’, ‘ARTool’, ‘patchwork’. Each box served as an independent replicate, ensuring data independence across treatments. We first conducted exploratory data analysis to examine distributions, identify potential outliers, and assess compliance with parametric assumptions. Normality of residuals was checked using the Shapiro–Wilk test, and homogeneity of variances was checked using the Levene test. When assumptions of ANOVA (normality and homogeneity of variances) were satisfied, we applied a two-way ANOVA. When assumptions were violated, we used the aligned rank transform (ART) ANOVA, which allows testing of factorial designs under nonparametric conditions while still requiring the independence of observations and correct specification of factors. The threshold of statistical significance was set at *p* < 0.05. Results are expressed as mean ± SE (Standard error).

## 3. Results

### 3.1. Survival

**Figure 1:**
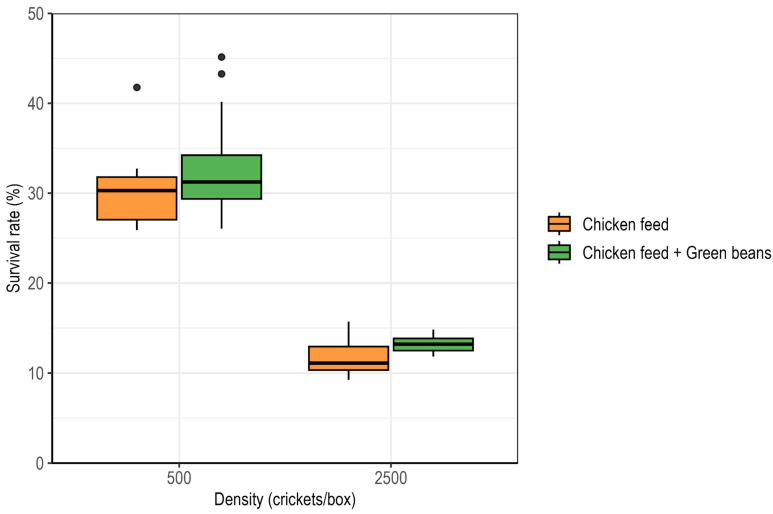
Effects of rearing density and green bean supplementation on survival in *Gryllus madagascarensis*. Survival is expressed as the proportion of individuals surviving to day 28.

Cricket survival differed significantly among treatments. Survival was significantly affected by rearing density (ART ANOVA; F_1,73_ = 220.83, *p* < 0.001) and diet (F_1,73_ = 17.17, *p* < 0.001), with no significant density × diet interaction (F_1,73_ = 0.39, *p* = 0.533). Survival was higher under low-density than under high-density conditions and increased with green bean supplementation at both densities. At low density, survivorship increased from 30.0 ± 0.9% under the control diet to 32.9 ± 1.2% with supplementation, while at high density it increased from 11.7 ± 0.4% to 13.2 ± 0.2% (Figure 1).

### 3.2. Chicken Feed Consumption

We examined whether supplementation of cricket feed with green beans decreased consumption of chicken feed under low and high-density rearing conditions.

**Figure 2:**
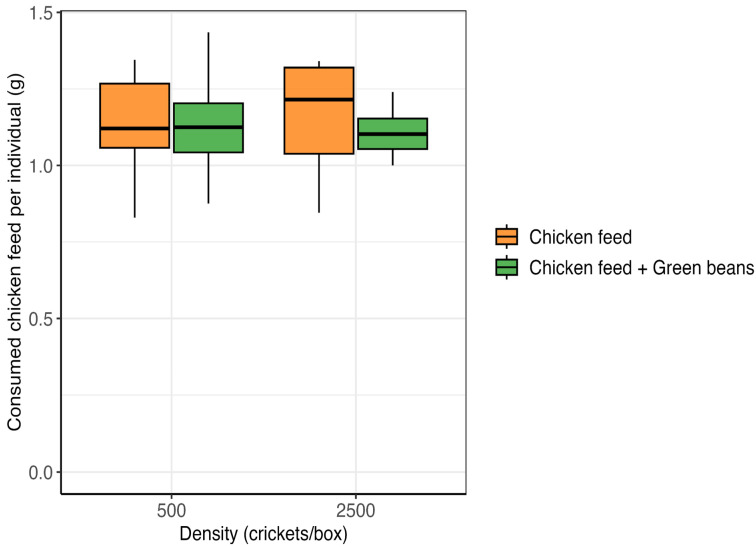
Effects of rearing density and green bean supplementation on chicken feed consumption in *Gryllus madagascarensis*. Values represent the median feed consumed per individual surviving cricket.

Chicken feed consumption was not significantly affected by density (ART ANOVA; F_1,73_ = 0.02, *p* = 0.885) nor diet (F_1,73_ = 2.75, *p* = 0.101), with no significant density × diet interaction (F_1,73_ = 0.55, *p* = 0.461). Chicken feed consumptions per surviving cricket were ranging from 1.1 ± 0.01 g to 1.17 ± 0.04 g (Figure 2).

### 3.3. Cricket Biomass Yield

**Figure 3:**
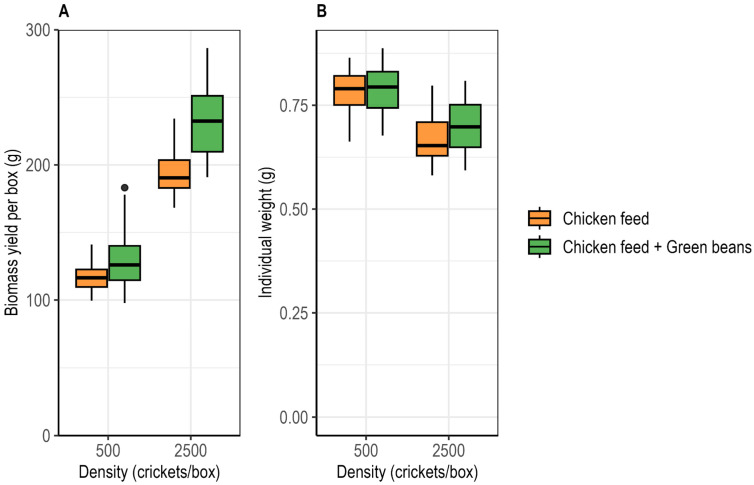
Effects of rearing density and green bean supplementation on biomass yield in *Gryllus madagascarensis*. (**A**) Total live biomass yield per box at day 28. (**B**) Median individual body weight at harvest.

Cricket biomass yield at day 28 differed significantly among treatments. On a total basis, yield per box was significantly affected by rearing density (ART ANOVA; F_1,73_ = 211.89, *p* < 0.001) and diet (F_1,73_ = 24.71, *p* < 0.001), with a significant density × diet interaction (F_1,73_ = 8.81, *p* = 0.0040). Yield per box was higher under high-density than under low-density conditions and increased with green bean supplementation at both densities. At low density, yield per box increased from 117.2 ± 2.5 g under the control diet to 129.7 ± 5.2 g with supplementation. At high density, yield per box increased from 194.4 ± 3.6 g to 231.2 ± 6.1 g (Figure 3A).

Biomass production per capita, i.e., body weight, was significantly affected by rearing density (two-way ANOVA; F_1,73_ = 54.10, *p* < 0.001) but not by diet (F_1,73_ = 1.04, *p* = 0.311), with no significant density × diet interaction (F_1,73_ = 0.70, *p* = 0.407). Individual body weight was higher under low-density than under high-density conditions. At low density, mean body weight was 0.784 ± 0.013 g under the control diet and 0.787 ± 0.013 g with supplementation, while at high density it was 0.672 ± 0.014 g and 0.697 ± 0.014 g, respectively (Figure 3B).

### 3.4. Frass Production

**Figure 4:**
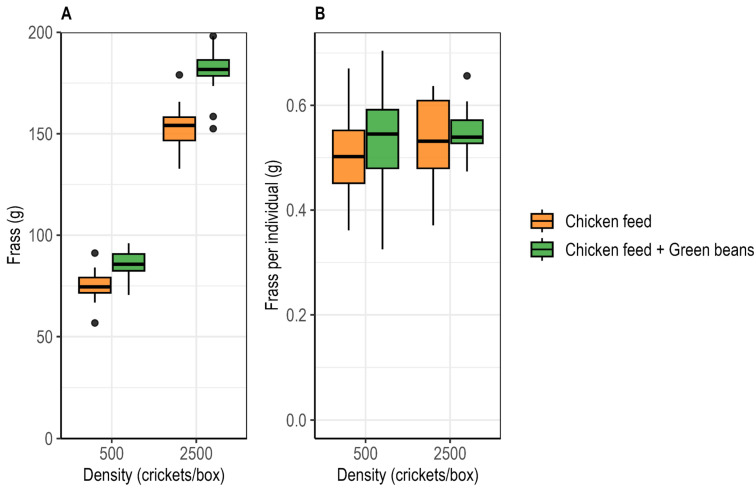
Effects of rearing density and green bean supplementation on frass production in *Gryllus madagascarensis*. (**A**) Cumulative frass production per box on day 28. (**B**) Median frass output per individual surviving cricket.

Frass production per box differed significantly among treatments. Frass production per box was significantly affected by rearing density (ART ANOVA; F_1,73_ = 219.28, *p* < 0.001) and diet (F_1,73_ = 75.76, *p* < 0.001), with a significant density × diet interaction (F_1,73_ = 25.23, *p* < 0.001). Frass production per box was higher under high-density than under low-density conditions and increased with green bean supplementation at both densities. At low density, frass output increased from 74.8 ± 1.7 g under the control diet to 84.9 ± 1.6 g with supplementation, while at high density it increased from 153.6 ± 2.8 g to 181.0 ± 2.4 g (Figure 4A).

Individual frass production was not significantly affected by density (ART ANOVA; F_1,73_ = 1.76, *p* = 0.189) nor diet (F_1,73_ = 0.74, *p* = 0.393), with no significant density × diet interaction (F_1,73_ = 0.43, *p* = 0.51). Per surviving cricket, frass production ranged from 0.51 ± 0.02 g to 0.55 ± 0.01 g (Figure 4B).

### 3.5. Feed Efficiency

#### 3.5.1. Efficiency of Conversion of Ingested Feed

**Figure 5:**
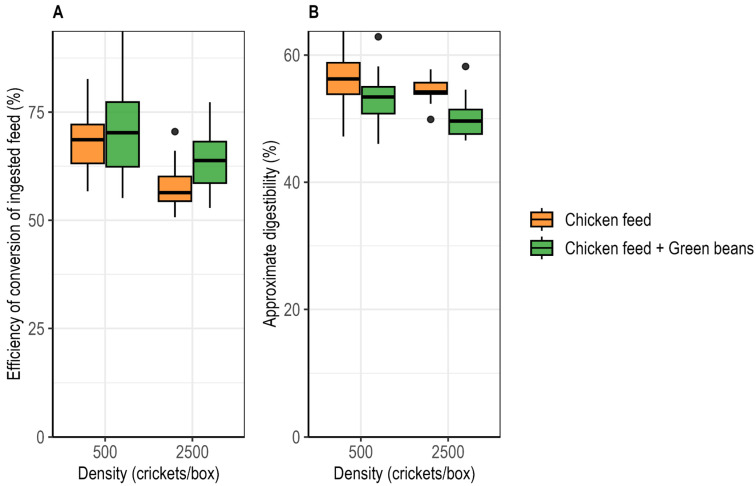
Effects of rearing density and green bean supplementation on feed efficiency in *Gryllus madagascarensis*. (**A**) Efficiency of conversion of ingested feed (ECI) per individual cricket. (**B**) Approximate digestibility (AD) per individual cricket.

Efficiency of conversion of ingested feed (ECI) differed significantly among treatments. ECI was significantly affected by rearing density (ART ANOVA; F_1,73_ = 29.65, *p* < 0.001) and diet (F_1,73_ = 5.25, *p* = 0.02), with no significant density × diet interaction (F_1,73_ = 1.22, *p* = 0.273). ECI was higher under low-density than under high-density conditions and increased with green bean supplementation at both densities. Under low-density conditions, ECI was 68.9 ± 1.7% under the control diet and 71.2% ± 2.3% with supplementation, while under high-density conditions it was 57.8 ± 1.2% and 63.6 ± 1.4% respectively (Figure 5A).

#### 3.5.2. Approximate Digestibility

Approximate digestibility (AD) was significantly affected by rearing density (ART ANOVA; F_1,73_ = 10.55, *p* = 0.001) and diet (F_1,73_ = 24.68, *p* < 0.001), with no significant density × diet interaction (F_1,73_ = 0.96, *p* = 0.329). Approximate digestibility was higher under low-density than under high-density conditions and decreased with green bean supplementation at both densities. Under low-density conditions, AD decreased from 56.0 ± 1.1% under the control diet to 53.2 ± 0.9% with supplementation, while under high-density conditions it decreased from 54.4 ± 0.4% to 50.2 ± 0.7% (Figure 5B).

## 4. Discussion

This study evaluated the effects of supplementing standard chicken feed with cooked green beans on survival, chicken feed intake, biomass yield, and frass production in *Gryllus madagascarensis* reared under contrasting stocking densities. Across all performance metrics, green bean supplementation consistently increased survival, biomass production, and frass production, while rearing density remained a dominant factor shaping individual performance and survival. Together, these results highlight how diet composition and density interact to determine productivity in intensive cricket farming systems.

### 4.1. Survival and Density-Dependent Mortality

Survival was strongly affected by rearing density, with markedly lower survival under high-density conditions. This pattern is consistent with density-dependent mortality observed in other crickets and orthopterans, where crowding increases stress, competition, and aggressive interactions [23,24,25]. In *Gryllus madagascarensis*, high-density mortality is likely driven in part by aggressive interactions and cannibalism, as reported in other cricket species.

Green bean supplementation improved survival at both densities, suggesting that the inclusion of a high-moisture feed mitigated some of the stressors associated with intensive rearing. Although crickets had continuous access to water throughout the experiment, the additional moisture provided by green beans may have contributed to reduced competition for water and lowered aggressive interactions. Comparable improvements in survival have been reported when moisture-rich supplements such as fresh leafy vegetables are added to cricket diets [26,27].

Although green beans added a small, transient increase in available surface immediately after feeding, they did not provide persistent structural complexity, and space limitations were not observed even at high density. Therefore, the observed improvements in survival are more likely attributable to nutritional supplementation rather than changes in surface area.

### 4.2. Chicken Feed Consumption

Supplementation of cricket feed with green beans did not reduce consumption of the commercial feed, indicating that crickets maintained stable per capita intake regardless of diet. Slight decreases in individual intake under higher density are consistent with density-dependent competition limiting access to resources, as reported in *Acheta domesticus* and other cricket species [28]. Overall, these findings suggest that individual feeding opportunities were largely stable across diets and densities.

### 4.3. Biomass Production and Growth Allocation

Cricket biomass yield increased with both stocking density and green bean supplementation. Higher density produced greater total biomass per box due to the larger number of individuals, despite lower survivorship and reduced individual body mass. Importantly, individual body weight was not significantly affected by diet, indicating that the increase in biomass under supplementation was driven primarily by improved survivorship rather than enhanced growth rates.

This pattern mirrors observations in other cricket species, where dietary supplementation increases final biomass largely through survival effects rather than changes in individual size [27]. These results emphasize that, under intensive rearing conditions, survivorship can be a more important determinant of total yield than individual growth performance.

### 4.4. Frass Production and Circularity

Frass production per box increased under higher rearing density and with green bean supplementation, while per capita frass production remained stable across treatments. This indicates that individual nutrient assimilation was largely unaffected by diet or density, even though total frass production rose.

From a circular-economy perspective, increased frass production represents an additional benefit of supplementation. Cricket frass is increasingly recognized as a valuable organic fertilizer that can improve soil fertility and crop performance [18,29]. Thus, higher frass output enhances the co-production of fertilizer alongside edible biomass, strengthening the integration of cricket farming into mixed crop–livestock systems.

### 4.5. Feed Conversion and Apparent Efficiency

Feed efficiency, calculated from per-surviving-cricket consumption of chicken feed, was influenced by both rearing density and diet. Efficiency of conversion of ingested feed (ECI) was higher under low-density conditions and increased with green bean supplementation, indicating that crickets were able to retain a greater proportion of consumed chicken feed as body mass. In contrast, approximate digestibility (AD) was slightly reduced with supplementation, likely reflecting the presence of indigestible components in the additional green beans, even though chicken feed intake itself remained unchanged. These findings suggest that green beans enhanced the conversion of chicken feed into biomass while modestly lowering apparent digestibility, and that crowding reduces both ECI and AD, likely due to density-dependent stress and competition.

### 4.6. Practical Implications for Cricket Farming

Together, these results provide clear guidance for cricket farming practice. Partial replacement of standard chicken feed with cooked green beans consistently increased survival and total biomass production, particularly under high-density conditions where crowding stress is most pronounced. For farmers with access to inexpensive or free green bean wastes, supplementation offers a practical strategy to increase yield per production unit without increasing dependence on commercial feed. The high moisture content of green beans may also reduce the need for separate water provisioning.

Stocking density should be selected based on production goals. Low-density rearing produced larger individual crickets and higher survivorship per egg, making it advantageous for markets targeting whole or premium crickets. In contrast, high-density rearing yielded greater total biomass per box despite smaller individual size and higher mortality, favoring production systems focused on powder or feed. Farmers should therefore adjust density according to whether their priority is individual size or total biomass output.

Finally, increased frass production under supplementation enhances the value of cricket farming within circular production systems, where both edible biomass and organic fertilizer contribute to overall farm productivity.

### 4.7. Limitations and Future Direction

This study also highlights several avenues for further research. The high moisture content of green beans complicates comparisons of feed efficiency on a fresh-weight basis, underscoring the need for future studies that standardize nutrient intake on a dry-matter basis. In addition, agro-industrial discarded materials such as green beans may vary in nutritional composition and microbial load across production batches, which could influence consistency at larger scales. Addressing these factors will be essential for translating experimental results into reliable commercial practice.

## 5. Conclusions

Supplementation of standard chicken feed with cooked green beans improved survival, feed conversion efficiency, biomass yield, and total frass production in *Gryllus madagascarensis* across rearing densities. These benefits appear to be primarily driven by nutritional supplementation rather than structural effects, as green beans provide additional moisture without creating persistent surface complexity. While individual body weight remained largely unchanged, higher survival and improved feed conversion resulted in greater total biomass production, particularly under high-density conditions. Rearing density remained a key determinant of performance, with high-density systems favoring bulk biomass and low-density systems favoring individual growth efficiency. As an abundant agro-industrial discarded material, cooked green beans offer a practical, low-cost supplement that can enhance productivity and strengthen the circularity of cricket farming systems.

## Figures and Tables

**Table 1:** Nutritional composition (%) of the two feeds (on a fresh-weight basis): chicken feed and cooked green beans.

	Chicken Feed	Cooked Green Beans
Moisture	9.33	90.8
Protein	21.5	1.04
Fat	2.93	<0.30
Fiber	6.62	3.20
Carbohydrate	53.1	3.39

## Data Availability

All data supporting the findings of this study are openly available in the GitHub repository “Supplementing cooked green beans on cricket diet” at https://github.com/Tahiry-Raharimandimby/Supplementing_cooked_green_beans_on_cricket_diet (accessed on 17 February 2026).

## Abbreviations

The following abbreviations are used in this manuscript:

ANOVA	Analysis of variance
ART ANOVA	Aligned rank transform analysis of variance
FCR	Feed conversion ratio
FA	Feed assimilation
SE	Standard error

## References

[1] OECD (2013). Global Food Security: Challenges for the Food and Agricultural System.

[2] United Nations (UN) Calls for Urgent Action to Feed the World’s Growing Population Healthily, Equitably and Sustainably. https://www.un.org/en/desa/un-calls-urgent-action-feed-world%E2%80%99s-growing-population-healthily-equitably-and-sustainably.

[3] Steinfeld H., FAO (2006). Livestock’s Long Shadow: Environmental Issues and Options.

[4] Gahukar R.T. (2016). Edible Insects Farming: Efficiency and Impact on Family Livelihood, Food Security, and Environment Compared with Livestock and Crops. Insects as Sustainable Food Ingredients.

[5] Jongema Y. (2017). Worldwide List of Recorded Edible Insects.

[6] Van Huis A., Van Itterbeeck J., Klunder H., Mertens E., Halloran A., Muir G., Vantomme P. (2013). Edible Insects: Future Prospects for Food and Feed Security.

[7] Van Itterbeeck J., Rakotomalala Andrianavalona I.N., Rajemison F.I., Rakotondrasoa J.F., Ralantoarinaivo V.R., Hugel S., Fisher B.L. (2019). Diversity and Use of Edible Grasshoppers, Locusts, Crickets, and Katydids (Orthoptera) in Madagascar. Foods.

[8] Magara H.J.O., Hugel S., Fisher B.L. (2024). Effect of Feed on the Growth Performance, Nutrition Content and Cost of Raising the Field Cricket (*Gryllus madagascarensis*) as a Sustainable Nutrient Source in Madagascar. Foods.

[9] Van Huis A. (2013). Potential of Insects as Food and Feed in Assuring Food Security. Annu. Rev. Entomol..

[10] Montowska M., Kowalczewski P.Ł., Rybicka I., Fornal E. (2019). Nutritional Value, Protein and Peptide Composition of Edible Cricket Powders. Food Chem..

[11] Oonincx D.G.A.B., Van Broekhoven S., Van Huis A., Van Loon J.J.A. (2019). Correction: Feed Conversion, Survival and Development, and Composition of Four Insect Species on Diets Composed of Food By-Products. PLoS ONE.

[12] Magara H.J.O., Niassy S., Ayieko M.A., Mukundamago M., Egonyu J.P., Tanga C.M., Kimathi E.K., Ongere J.O., Fiaboe K.K.M., Hugel S. (2021). Edible Crickets (Orthoptera) Around the World: Distribution, Nutritional Value, and Other Benefits—A Review. Front. Nutr..

[13] Oonincx D.G.A.B., De Boer I.J.M. (2012). Environmental Impact of the Production of Mealworms as a Protein Source for Humans—A Life Cycle Assessment. PLoS ONE.

[14] Lundy M.E., Parrella M.P. (2015). Crickets Are Not a Free Lunch: Protein Capture from Scalable Organic Side-Streams via High-Density Populations of *Acheta domesticus*. PLoS ONE.

[15] Morales-Ramos J.A., Rojas M.G., Dossey A.T., Berhow M. (2020). Self-Selection of Food Ingredients and Agricultural by-Products by the House Cricket, *Acheta domesticus* (Orthoptera: Gryllidae): A Holistic Approach to Develop Optimized Diets. PLoS ONE.

[16] Ojha S., Bußler S., Schlüter O.K. (2020). Food Waste Valorisation and Circular Economy Concepts in Insect Production and Processing. Waste Manag..

[17] Annrose W., Peter B., Darius A. (2023). Effects of Cricket Frass on Vegetative Growth of Cleome Gynandra. Afr. J. Agric. Res..

[18] Andrianorosoa Ony C., Solofondranohatra C.L., Ramiadantsoa T., Ravelomanana A., Ramanampamonjy R.N., Hugel S., Fisher B.L. (2024). Effect of Cricket Frass Fertilizer on Growth and Pod Production of Green Beans (*Phaseolus vulgaris* L.). PLoS ONE.

[19] Magara H.J.O., Solofondranohatra C.L., Hugel S., Fisher B.L. (2025). Weeds and Agro By-Products for Sustainable Farming of Edible Field Cricket, *Gryllus madagascarensis* (Orthoptera: Gryllidae). PLoS ONE.

[20] ANSES Ciqual Table de Composition Nutritionnelle Des Aliments. https://ciqual.anses.fr/#/aliments/20502/haricot-bean-boiled-cooked-in-water.

[21] Waldbauer G.P. (1968). The Consumption and Utilization of Food by Insects. Advances in Insect Physiology.

[22] R Core Team (2024). R: A Language and Environment for Statistical Computing.

[23] Tennis P.S., Koonce J.F., Teraguchi M. (1977). The Effects of Population Density and Food Surface Area on Body Weight of *Acheta domesticus* (L.) (Orthoptera: Gryllidae). Can. J. Zool..

[24] Jonsson T. (2017). Metabolic Theory Predicts Animal Self-thinning. J. Anim. Ecol..

[25] Mahavidanage S., Fuciarelli T.M., Li X., Rollo C.D. (2023). The Effects of Rearing Density on Growth, Survival, and Starvation Resistance of the House Cricket *Acheta domesticus*. J. Orthoptera Res..

[26] Mazurkiewicz A., Tumialis D., Pezowicz E., Urbaēski J., Galewski P., Góral K. (2013). The Effect of Density on the Breeding Optimization of the Tropical House Cricket Gryllodes Sigillatus (Walker) (Orthoptera: Gryllidae). Ann. Wars. Univ. Life Sci.-SGGW.

[27] Pomari Fernandes A., Cazarolli L.H., Pigatto T., Trento E., Retcheski M.C., Quast L.B., Romão S., Tormen L., Pinto V.Z. (2025). Exploring Side Streams Upcycling for Crickets Farming: Insects Biology and Chemical Composition. Food Biosci..

[28] Gutiérrez Y., Fresch M., Ott D., Brockmeyer J., Scherber C. (2020). Diet Composition and Social Environment Determine Food Consumption, Phenotype and Fecundity in an Omnivorous Insect. R. Soc. Open Sci..

[29] Beesigamukama D., Subramanian S., Tanga C.M. (2022). Nutrient Quality and Maturity Status of Frass Fertilizer from Nine Edible Insects. Sci. Rep..

